# Neural Epidermal Growth Factor-Like Like Protein 2 (*NELL2*) Promotes Aggregation of Embryonic Carcinoma P19 Cells by Inducing N-Cadherin Expression

**DOI:** 10.1371/journal.pone.0085898

**Published:** 2014-01-21

**Authors:** Dong Hee Kim, Han Rae Kim, Eun Jung Choi, Dong Yeol Kim, Kwang Kon Kim, Byung Sam Kim, Jeong Woo Park, Byung Ju Lee

**Affiliations:** Department of Biological Sciences, College of Natural Sciences, University of Ulsan, Ulsan, South Korea; University of Freiburg, Germany

## Abstract

NELL2 was first identified as a mammalian homolog of chick NEL (Neural EGF-like) protein. It is almost exclusively expressed in neurons of the rat brain and has been suggested to play a role in neural differentiation. However, there is still no clear evidence for the detailed function of NELL2 in the differentiation of neurons. In this study, we identified NELL2 function during neural differentiation of mouse embryonic carcinoma P19 cells. Endogenous expression of NELL2 in the P19 cells increased in parallel with the neuronal differentiation induced by retinoic acid (RA). We found that the mouse *NELL2* promoter contains RA response elements (RAREs) and that treatment with RA increased *NELL2* promoter activity. Transfection of P19 cells with *NELL2* expression vectors induced a dramatic increase in cell aggregation, resulting in the facilitation of neural differentiation. Moreover, NELL2 significantly increased N-cadherin expression in the P19 cell. These data suggest that NELL2 plays an important role in the regulation of neuronal differentiation via control of N-cadherin expression and cell aggregation.

## Introduction

The secreted N-glycosylated protein, NELL2, is specifically expressed in neural tissues [1]–[3]. NELL2 contains a signal peptide and multiple functional domains such as an N-terminal thrombospondin-1-like domain, six epidermal growth factor-like domains, and five von Willebrand Factor C-like domains. Thus, NELL2 has been suggested to play multifunctional roles in the proliferation and differentiation of neural cells and as a possible trophic factor [1], [4], [5].

Involvement of NELL2 in neural cell differentiation has been proposed because its expression is closely correlated with neurogenesis and differentiation of the neural cells during development [3], [4], [6], and it is localized to the site of hippocampal adult neurogenesis [7]. Moreover, NELL2 expression is maximized during the peak period of neurogenesis and differentiation of both spinal cord motor neurons and sensory neurons within the dorsal root ganglia [6]. It was reported that NELL2 drives neuroprogenitor cells to exit the cell cycle and promotes their precocious differentiation, and increases the rate of motor neuron differentiation in the spinal cord motor pools [8].

However, the details of NELL2 function in the early stage of neural differentiation remain unclear. Interestingly, NELL2 expression is increased in mouse embryonic stem cells when they are induced to differentiate into neurons in response to retinoic acid (RA) [9]. RA is an important cue for regulating differentiation of neuroprogenitor cells [10]. Many functions of RA are mediated by the RA-induced transcriptional regulation of various genes via binding with two distinct receptors, the RA receptors (RARs) and retinoid X receptors (RXRs) [11], [12]. The *NELL2* promoter contains presumptive half RAR/RXR binding domains [13]. Therefore, RA with its receptor(s) may regulate *NELL2* gene expression through binding to these sites.

The role of RA in neuronal differentiation of the nervous system has been studied extensively using an *in vitro* model such as embryonic carcinoma P19 cells. Treatment of aggregated P19 cells with higher concentration (greater than 0.5 µM) of RA results in differentiation into neurons and glia [10], [14], [15] by activating the transcription of many genes, including those encoding transcription factors, cell signaling molecules, structural proteins, enzymes and cell-surface receptors [16]. Therefore, the RA-induced differentiation of P19 cells provides a useful model for identification and characterization of factors that regulate neuronal differentiation and development [17].

In this study, we have investigated a possible role for NELL2 in the neuronal differentiation of P19 cells. For the induction of neuronal differentiation, P19 cells were allowed to aggregate for 4 days in the presence of RA and were replated for 4 days without RA. Here, we demonstrate that RA strongly induced P19 cells to express NELL2, resulting in aggregation and differentiation of cells into a neuronal phenotype.

## Materials and Methods

### Cell culture and Transfection of *NELL2* expression vectors

P19 embryonic carcinoma cells were obtained from American Type Culture Collection (ATCC, Catalogue No. CRL-1825) and cultured in α-modified Eagle's medium (α-MEM, Hyclone, South Logan, UT), supplemented with 10% fetal bovine serum (FBS) and 100 U/ml penicillin-streptomycin (Hyclone) under a humidified atmosphere with 5% CO_2_ in air at 37°C. For stable transfection, P19 cells were transfected with pcDNA-DEST40 control vector (Invitrogen Corp., Carlsbad, CA) or the pcDNA-NELL2 expression vector that encodes the *NELL2* gene by using Lipofectamine/PLUS reagent (Invitrogen). The transfected P19 cells were selected in the presence of the G418 (400 µg/ml, Sigma-Aldrich, ST. Louis, MO) for 3 weeks, and the medium was changed every 2 days. The G418-resistant clones were harvested and analyzed by reverse transcription (RT)-PCR and Western blot.

### Induction of neuronal differentiation

To induce neural differentiation of P19 cells, the cells were allowed to aggregate in bacteriological grade petri dishes at a seeding density of 1×10^6^ cells/ml in the presence of 1 µM all-trans-retinoic acid (RA, Sigma-Aldrich) in α-MEM with 5% FBS, as previously described [18]. After 4 days of aggregation, the cells were harvested using a Cell Strainer (SPL Life Science, Pocheon, Korea) and dissociated into single cells using a 0.25% trypsin-EDTA (Hyclone) solution, and then were replated in poly-L-lysine (Sigma-Aldrich)-coated tissue culture dishes at a density of 1×10^4^ cells/ml. The cells were allowed to adhere and were cultured in the absence of RA for 4 days. To determine involvement of extracellular signal-regulated kinase (ERK) signaling in the NELL2-induced aggregation of P19 cells, the cells were incubated with mitogen-activated protein kinase kinase (MEK) inhibitor, U0126 (5 µM, Calbiochem, San Diego, CA) during the aggregation period.

### Cell aggregation assays

P19 cells permanently transfected with NELL2 expression vectors were transferred at a density of 1×10^6^ cells/ml on 100-mm plates for 2 and 4 days in the presence of 1 µM RA or dimethyl sulfoxide (DMSO, final concentration of 1.0×10^−5^ µl/ml) in α-MEM containing 5% FBS. The number of single cells was counted, and the percentage of single cells during the aggregation assay was determined by the index N_d_/N_0_, where N_d_ is the total number of single cells after a certain incubation day, and N_0_ is the total number of single cells at initiation of incubation [19].

### Site-directed mutagenesis and promoter assays

Site-directed mutagenesis was used to mutate each of the two half-RA response elements (RAREs) identified in the mouse *NELL2* (m*NELL2*) promoter (Fig. S1). The mutant promoters were generated from wild-type m*NELL2* promoter [13] as the template. Mutagenesis was carried out using QuickChange-XLsite-directed mutagenesis kit (Stratagene, La Jolla, CA) according to the manufacturer's instructions. The primers used for mutagenesis were as follows: a primer set for deleting one half-RARE, 5′-GAA TCC CCT TGC CTT GCC CTT TTG CTG CTG TGT AG-3′ and its complementary sequence (CS), located at the *NELL2* promoter (−1047); a set for deleting another half-RARE, 5′-GTC CCC GCA GGT CCC CAG AGC CGG CTG CGG CC-3′ and its CS, located at −223 of the *NELL2* promoter. Mutants were verified by DNA sequencing. To determine whether RA regulates NELL2 transcription, the P19 cells were transiently transfected with m*NELL2* promoter (NELL2-P)-luciferase reporter constructs (NELL2-pGL3) using Lipofectamine/PLUS reagent. After 24 h, the cells were treated with 1 µM RA for 24 h and lysed with Cell Lysis Reagent (Promega Corp., Madison, WI). Luciferase assays were performed using a luciferase reporter assay kit (Promega Corp.). The transfection efficiency of each assay was normalized by cotransfecting the plasmid p-CMV-β-gal (Clontech, Palo Alto, CA) at 60 ng/ml.

### RNA interference

For knocking down endogenous synthesis of NELL2 in P19 cells, a small interference RNA (siRNA) was constructed by automated solid phase synthesis (Bioneer Primer Synthesis Service, Daejeon, Korea) and was transfected into the P19 cells using the Lipofectamine/PLUS reagent. The siRNA duplex used for targeting m*NELL2* mRNA was composed of sense (5′-GGA CGA AAG CCU UCC UCU UCC-3′) and antisense (5′-AAG AGG AAG GCU UUC GUC CAC-3′) sequences. The negative control siRNA duplex consisted of sense [5′- CCU ACG CCA CCA AUU UCG (dTdT)-3′] and antisense [5′-ACG AAA UUG GUG GCG UAG G (dTdT)-3′] sequences (Bioneer).

### RNA isolation and quantitative real-time PCR

To determine the effect of RA on endogenous NELL2 expression in P19 cells and to investigate the effect of RA and/or NELL2 on the expression of neuronal markers, RNA (2 µg) isolated form P19 cells (control cells or cells permanently transfected with NELL2 expression vectors) treated with RA (1 µM) was reverse-transcribed and amplified by real-time PCR using the primer sets shown in Table S1. Real-time PCR reactions [20 µl total volume containing 5 pmol of primer, 10 µl of SYBR Green dye (Qiagen, Valencia, CA), and 2 µl of cDNA] were carried out with a DNA Engine Opticon Continuous Fluorescence Detection System (MJ Research, Inc., Waltham, MA) for 40 cycles.

### Western blotting

Proteins from P19 cells were homogenized in M-PER lysis buffer (Pierce Chemical Co., Rockford, IL). Extracted proteins (15 µg) were separated by SDS-polyacrylamide gel electrophoresis and were electrophoretically transferred onto a membrane according to the previously described approach [20]. The membrane was blocked in blocking buffer and incubated with antibodies to NELL2 (Santa Cruz Biotech., Santa Cruz, CA, Catalogue No. sc-54637), β-actin (Sigma-Aldrich, Catalogue No. A5441), Tuj1 (Santa Cruz Biotech., Catalogue No. sc-5274), NeuN (Millipore, Billerica, MA, Catalogue No. MAB377), N-cadherin (Abcam, Boston, MA, Catalogue No. ab76057), phosphorylated ERK (pERK) (CELL Signaling Technology, Beverly, MA, Catalogue No. 9101), ERK (Santa Cruz Biotech., Catalogue No. sc-153), or c-Fos (Santa Cruz Biotech., Catalogue No. sc-7202). Blots were developed using horseradish peroxidase-conjugated anti-goat secondary antibody (Santa Cruz Biotech., Catalogue No. sc-2020), anti-mouse secondary antibody (Santa Cruz Biotech., Catalogue No. sc-2005) or anti-rabbit secondary antibody (Santa Cruz Biotech., Catalogue No. sc-2004). Immunoreactivity was detected with an enhanced chemiluminescence (ECL) kit (Amersham Pharmacia Biotech., Buckinghamshire, UK).

### Electrophoretic Mobility Shift Assays (EMSAs)

To perform EMSA, P19 cells were incubated with 1 µM RA for 24 h, and nuclear protein extracts were prepared according to the previously described method [21] utilizing a protease inhibitor cocktail [22]. The double-stranded oligodeoxynucleotide probes were end-labeled with [γ-^32^P] ATP and purified over a NICK column (Bio Rad Laboratories, Hercules, CA). The binding assay was performed as described [13] with minor modifications using 5 µg nuclear protein extracts, 20,000 cpm probe and 1 µg poly (dI-dC). The reaction mixtures were separated by electrophoresis on a 6% non-denaturing polyacrylamide gel. The gels were then dried and exposed to film at −80°C.

The oligodeoxynucleotide probes for EMSA were as follows: a positive control probe (5′-AGG GTA GGG TTC ACC GAA AGT TCA CTC-3′) (Santa Cruz Biotech., Catalogue No. sc-2559) containing the palindromic RARE and its flanking sequence [23]; a negative control probe (5′-AGG GTA GGG AAC ACC GAA AGT TCA CTC-3′) (Santa Cruz Biotech., Catalogue No. sc-2560) containing a mutation in one arm of the palindromic sequence of the positive control probe [24]; a probe containing the half-RARE sequence (5′-GAG AGC CTG ACC CGG CTG C-3′) located at the -223 site of m*NELL2* promoter; a probe containing the other half-RARE sequence (5′-TGC CCT TTG ACC CTT GCT G-3′) located at the −1047 site of the promoter; and another negative control probe (5′-AGG CCG CCC CGC CCG CGC C-3′) located at the −460 of the promoter.

### Chromatin immunoprecipitation (ChIP) assays

ChIp assays were performed using nuclei extracted from the P19 cells treated with RA, as previously described in detail [13]. Briefly, chromatin mixture diluted in ChIP dilution buffer (0.01% SDS, 1.1% Triton X-100, 1.2 mM EDTA, 16.7 mM Tris, pH 8.1, 167 mM NaCl, and protease inhibitors) was incubated with 5 µg of rabbit polyclonal antibodies against human RAR (Santa Cruz Biotech., Catalogue No. sc-773) at 4°C overnight. DNA from the protein-DNA cross-links was extracted from the immune complexes and was further purified with phenol/chloroform. PCR amplification was performed using 35 cycles of 94°C for 30 s, 53°C for 30 s and 72°C for 30 s, proceeded by 94°C for 5 min, and followed by 72°C for 10 min. Primer sets used for the PCR amplification included a primer set for one half-RARE at -223 (sense primer, 5′-CCT CCC TTC CTC TGC GTG-3′; antisense primer, 3′-CAC CTA AGA CCG AGC GGG -5′), and a primer set for the other half-RARE at -1047 (sense 5′-ATC CAT CCA TCC ATC CAT CC-3′; antisense, 3′-TGC TGG ACA GCT CCA GAA AC-5′), and a primer set for the negative control site at -460 (sense 5′-GTG AGG GCT TCC CTC TTT TG-3′; antisense, 3′-GTC TCC AGG AGT TGG TGG GA -5′).

### Statistics

Student's t-test was used to determine the significance of difference between control and experimental groups. P values <0.05 were considered to be statistically significant.

## Results

### RA increases mNELL2 promoter activity and levels of endogenous mRNA and protein in P19 cells

**Figure 1:**
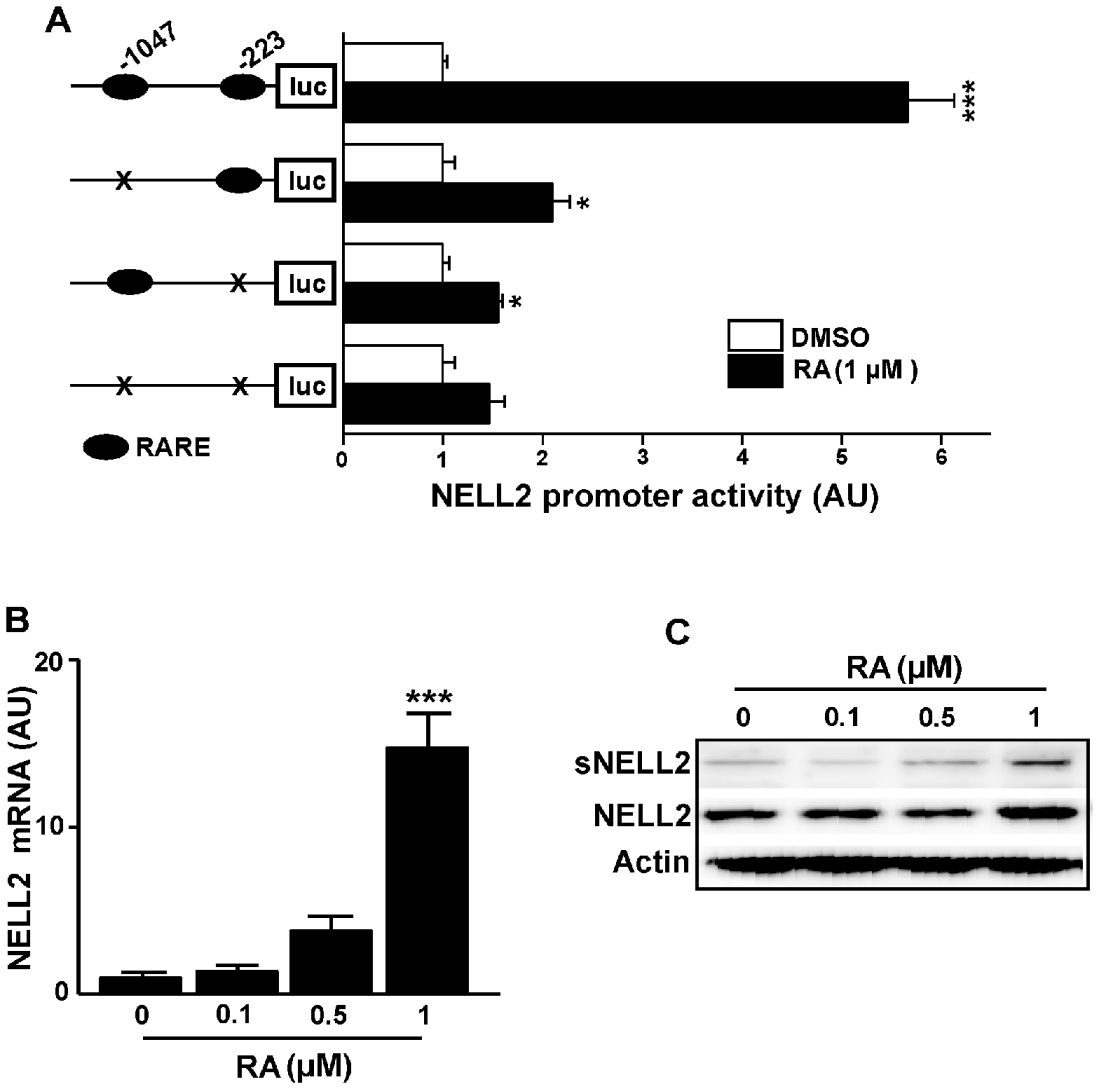
Retinoic acid (RA) activates m*NELL2* promoter activity and expression of endogenous *NELL2* mRNA and protein in P19 cells. (A) To determine the effects of RA on m*NELL2* promoter activity, a 1.3 kb m*NELL2* promoter-luciferase (luc) reporter construct was transfected into the P19 cells, and the luciferase activity was determined 24 h after the RA treatment. (B) Real-time PCR analysis showing the change in endogenous *NELL2* mRNA expression by RA treatment. (C) Western blot analysis to determine changes in RA-induced NELL2 expression. High concentration (1 µM) of RA increased intracellular NELL2 or secreted NELL2 (sNELL2). All experiments were repeated at least four times and data are presented as mean ± SEM. *, p<0.05; ***, p<0.001 versus control (DMSO for A, 0 µM for B).

To analyze the effect of RA on the NELL2 transcriptional activity, we used the m*NELL2* promoter construct [13]. This promoter sequence contains two half-RAREs at −1047 and at −223 upstream from the translational start site (Fig. S1). As shown in Fig. 1A, RA (1 µM) strongly activates *NELL2* promoter activity in P19 cells. To determine whether the two half-RAREs are essential to the RA-induced stimulation of the *NELL2* promoter activity, we deleted each of both half-RARE motifs by site-directed mutagenesis and examined the ability of RA to transactivate the mutant promoter in P19 cells. As shown in Fig. 1A, deletion of each half-RARE resulted in a significant decrease in promoter activity induced by RA.

Real-time PCR analysis revealed that treatment with RA induced a dose-related increase in *NELL2* mRNA, and high concentration (1 µM) of RA caused a significant increase in the endogenous *NELL2* mRNA abundance in P19 cells (Fig. 1B). In addition to the pattern of change in mRNA level, Western blotting revealed that 1 M RA induced an increase in NELL2 protein from the cell extracts as well as in the media (Fig. 1C).

### RAR binds to half-RARE domains in the 5′-flanking region of the *NELL2* gene

**Figure 2:**
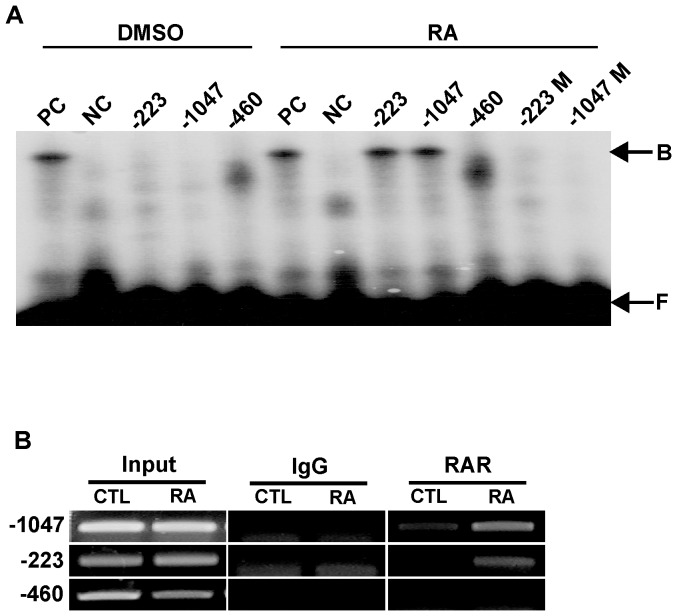
EMSA and ChIP assays. EMSAs were performed using double-stranded oligomer probes containing the putative half-RAREs found in the m*NELL2* promoter sequence. (A) Autoradiogram showing binding activity of half-RAREs derived from the m*NELL2* promoter to the nuclear extracts from P19 cells treated with RA (1 µM). Numbers on the gel images indicate the sites in the m*NELL2* promoter sequence where the oligomer probes were designed; M following the numbers means that the indicted probes bear a mutation in the half-RARE sites. B, protein-bound DNA; F, free DNA; PC, a positive control palindromic RARE; NC, a negative control of mutant RARE. (B) ChIP assays using DNA precipitated by using RAR antibodies. The immunoprecipitated DNA from P19 cells treated with RA or DMSO was PCR-amplified using primer sets designed to detect m*NELL2* promoter fragments including the two half-RARE sequences (at −223 and −1047). Input represents the used DNA extracted from the P19 cells before immunoprecipitation. Normal rabbit IgG was included for immunoprecipitation in the assay as a negative control.

To determine whether RAR binds to each of the two half-RARE domains present in the *NELL2* promoter, we performed EMSAs and ChIP assays. For the EMSAs, we used oligomer probes containing the core half-RARE motif (5′-TGACCC-3′) at −1047 and −223, and their flanking sequences (Fig. S1). Both probes resulted in shifted bands when exposed to nuclear extracts from P19 cells treated with RA (Fig. 2A). A positive control probe containing a palindromic RARE (labeled as PC) revealed a band of an apparently similar size. In contrast, a mutated palindromic RARE (NC), mutated half-RARE at −1047 (M) and −223 (M) and a negative control sequence at −460 did not generate any specific band.

To further determine the *in vivo* interaction of RAR with the two half-RARE sequences of the *NELL2* promoter, ChIP assays were performed using RAR antibody and precipitated DNA was amplified using PCR primers specific to the promoter regions containing the half-RARE. As shown in Fig. 2B, DNA fragments immunoprecipitated with RAR antibody generated a specific band by amplification with primer sets; one primer set amplified a 183-bp fragment of the *NELL2* promoter region of −307 to −124 encompassing the half-RARE at −223, while the other primer set amplified a 209-bp fragment corresponding to the −1183 to −974 region of the *NELL2* promoter with the half-RARE at −1047. However, a negative control primer set for the sequence surrounding −460 did not generate any positive band. The results reveal that the PCR fragment containing the half-RARE sequences at −223 and −1047 were markedly increased in DNA samples from the RA-treated P19 cells compared with DNA samples from control cells, suggesting that the RA-RAR complex strongly binds to the two half RARE sequences.

### Expression of NELL2 mRNA during neuronal differentiation of P19 cells

**Figure 3:**
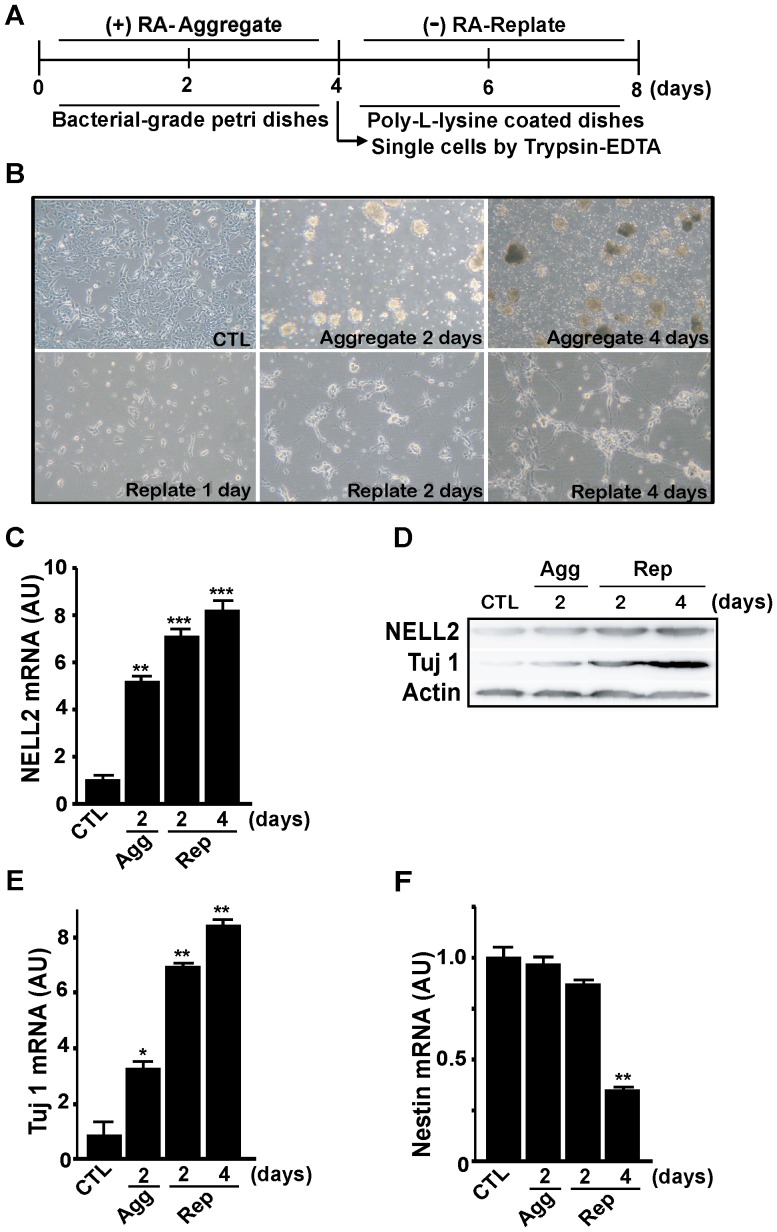
Change in NELL2 expression during the RA-induced neuronal differentiation of P19 cells. (A) General scheme for the neuronal differentiation process in this study. P19 cells were aggregated for 4 days with 1 µM RA treatment, and the aggregates were harvested and replated as the single-cell suspension and cultured without RA for 4 days. (B) Representative photos showing morphological changes in P19 cells during the neuronal differentiation process. Cells show aggregated morphologies as embryonic bodies at 2 and 4 days of aggregation, and reveal bipolar shapes with processes at 2 and 4 days after replating. Control (CTL) represents 2 days of aggregation without any treatment. (C, E, F) Real-time PCR analysis of *NELL2* (C), *Tuj-1* (E) and *Nestin* (F) mRNA expression in the P19 cells during the process of aggregation and replating. Agg, aggregation; Rep, replating; AU, arbitrary units. All experiments were repeated at least four times and data are presented as mean ± SEM. *, p<0.05; **, p<0.01; ***, p<0.001 versus control (CTL). (D) Western blot analysis of NELL2 and Tuj-1 protein expression during the neuronal differentiation process of the P19 cells.

To induce neuronal differentiation of murine P19 embryonic carcinoma cells, the cells were cultured as free-floating embryonic bodies (EBs) in defined medium containing 1 µM RA. After 4 days incubation with RA, EBs were digested into single cells and plated onto the culture dishes without RA (Fig. 3A). Extensive morphological changes indicating neuronal differentiation began 2 days after replating and became clearer 4 days after replating (Fig. 3B). During neuronal differentiation of the cells treated with RA and aggregation and replating, there was a dramatic increase in *NELL2* mRNA expression (Fig. 3C) and protein levels (Fig. 3D). During the neuronal differentiation process, the cells expressed an increased amount of mRNA for the neuronal marker Tuj1 (Fig. 3E), while mRNA expression of the neuroprogenitor marker Nestin was significantly decreased at 4 days after replating (Fig. 3F).

### *NELL2* promotes the cellular aggregation and neuronal differentiation of P19 cells

**Figure 4:**
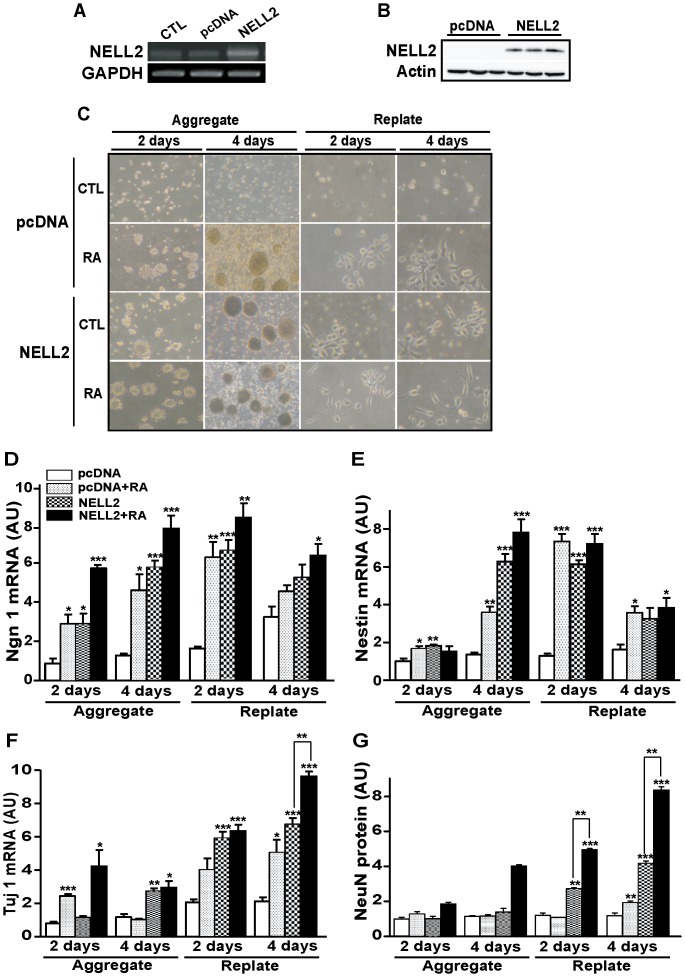
NELL2 promotes neuronal differentiation of P19 cells. (A) Overexpression of NELL2 in P19 cells permanently transfected with NELL2 expression vectors confirmed by RT-PCR using RNA extracted from the cells. CTL, control cells; pcDNA, cells transfected with control pcDNA vectors; NELL2, cells transfected with NELL2 expression vectors. (B). Western blot analysis for overexpression of NELL2 protein in the P19 cells permanently transfected with NELL2 expression vectors. (C) Representative photograms showing the morphological changes of P19 cells by overexpression of the NELL2 expression vectors with or without the treatment of RA (1 µM). (D–F) Real-time PCR analysis of *Ngn-1* (D), *Nestin* (E) and *Tuj-1* (F) mRNA expression in the P19 cells overexpressing NELL2 during the aggregation and replating process of the neuronal differentiation. RNA samples were collected from P19 cells with the indicated treatment at 2 and 4 days after aggregation and replating. (G) Western blot analysis of NeuN protein expression in P19 cells during aggregation and replating processes. All experiments were repeated at least four times and data are presented as mean ± SEM. *, p<0.05; **, p<0.01; ***, p<0.001 versus control pcDNA group.

To investigate the role of NELL2 during the neuronal differentiation of P19 cells, we examined the morphology and expression of neuronal markers in cells stably transfected with NELL2 expression vector. RT-PCR and Western blot analyses revealed that the stably transfected NELL2 effectively increased expression of NELL2 (Fig. 4A and B). Morphological changes of these cells were observed during neuronal differentiation after RA treatment. Interestingly, cells overexpressing NELL2 began to form EBs at 2 days of aggregation (indicated as aggregate in Fig. 4C) without RA treatment and further displayed neuron-like morphologies with the bipolar structure protruding processes at 2 and 4 days after segregation into single cells and replating (shown in Fig. 4C). These results are similar to those observed in the RA-treated group (Fig. 4C). However, no clear change was observed in the control treated cells transfected with control pcDNA vectors, during the same period of neuronal induction. These results suggest that overexpression of NELL2 in P19 cells is sufficient to trigger cellular aggregation and neuronal differentiation of P19 cells in the absence of RA.

The effect of NELL2 on the neuronal differentiation of the P19 cells was further confirmed by determining expression of several marker genes for neuronal differentiation, such as *Ngn 1*, *Nestin*, *Tuj-1* and *NeuN* [25], [26]. Generally, mRNA levels of the neuroprogenitor markers such as *Ngn 1* and *Nestin* began to increase at 2 days of aggregation and reached a peak at 2 days after replating (Fig. 4D and E). Interestingly, NELL2 overexpression alone induced an increase in *Ngn 1* (Fig. 4D) and *Nestin* (Fig. 4E) mRNA levels, compared to the pcDNA-transfected control group at most stages of neuronal differentiation. Moreover, the *Ngn 1* and *Nestin* mRNA levels in the NELL2 group were similar to those in the RA-treated pcDNA-transfected control cells at most phases. Compared to the neuroprogenitor markers, expression of the mRNA for the neuronal marker Tuj1 and NeuN protein dramatically increased at 2 and 4 days after replating (Fig. 4F and G). Importantly, NELL2 alone induced an increase of not only *Tuj1* mRNA (Fig. 4F) during the neuronal differentiation processes after 4 days of aggregation, but also NeuN protein levels (Fig. 4G) at 2 and 4 days after replating, compared to the control pcDNA and/or RA-treated pcDNA groups. Furthermore, RA treatment to the cells overexpressing NELL2 further increased the *Tuj1* mRNA level at 4 days after replating and NeuN protein contents at 2 and 4 days after replating compared to the NELL2 overexpressing group, respectively, suggesting that some other factor(s) induced by the RA treatment may also contribute to the expression of Tuj1 and NeuN at these points, respectively. These data together suggest that NELL2 may facilitate the process underlying neuronal differentiation of the P19 cells.

### Effect of *NELL2* on P19 cell aggregation

**Figure 5:**
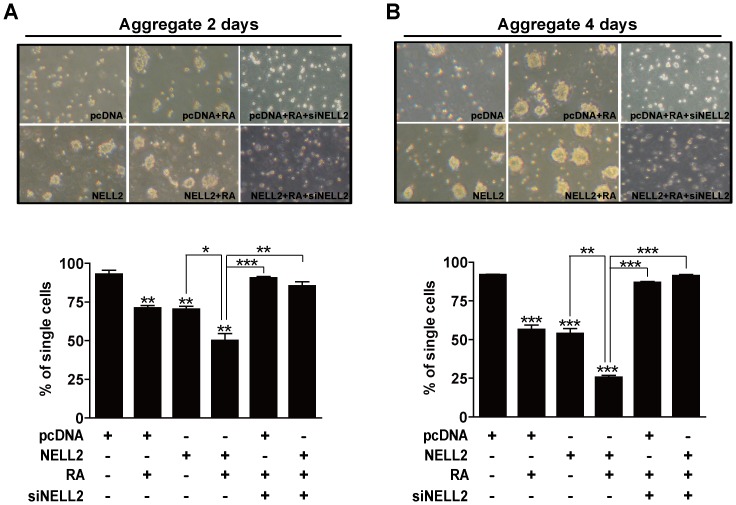
NELL2 promotes aggregation of P19 cells. P19 cells permanently transfected with NELL2 expression vectors were cultured in the presence or absence of 1 µM RA for 2 (A) or 4 (B) days. To knock down NELL2 synthesis, the indicated groups of P19 cells were transfected with siRNA against *NELL2* mRNA (siNELL2). Each upper panel reveals representative photos and the lower panel includes results showing difference in single cell numbers among treatment groups. All experiments were repeated at least four times and data are presented as mean ± SEM. *, p<0.05; **, p<0.01; ***, p<0.001 versus control pcDNA group or between lined groups.

Because the most prominent change in the cells due to overexpression of NELL2 was their autonomic aggregation and neurosphere formation without RA treatment, we further examined if the transfection of NELL2 indeed stimulated cell aggregation by using cell aggregation assays. As indicated in Fig. 5, the group overexpressing NELL2 resulted in significantly lower single cell numbers at 2 (Fig. 5A) and 4 (Fig. 5B) days after floating culture compared to the control pcDNA transfected group, and showed similar single cell numbers with those of the RA-treated pcDNA group. RA treatment of the NELL2 overexpressing cells further decreased the number of single cells. Therefore, NELL2 is likely to be involved in cell aggregation at the beginning of the neuronal differentiation process.

To further confirm the effect of NELL2 on the aggregation of P19 cells, the cells were observed after knocking down synthesis of NELL2 with *NELL2* siRNA, as previously described [13]. Transfecting the cells with *NELL2* siRNA resulted in almost complete inhibition of endogenous NELL2 expression in the P19 cells (data not shown), as shown in a previous report [13]. *NELL2* siRNA decreased the aggregate size as shown in Fig. 5A and B, and vice versa, increased single cell numbers similar to that of the control that received neither RA treatment nor NELL2 transfection. On the contrary, transfection with a control siRNA that does not interfere with the NELL2 synthesis neither decreased the aggregate size nor increased single cell numbers (data not shown).

### *NELL2* increases N-cadherin expression

**Figure 6:**
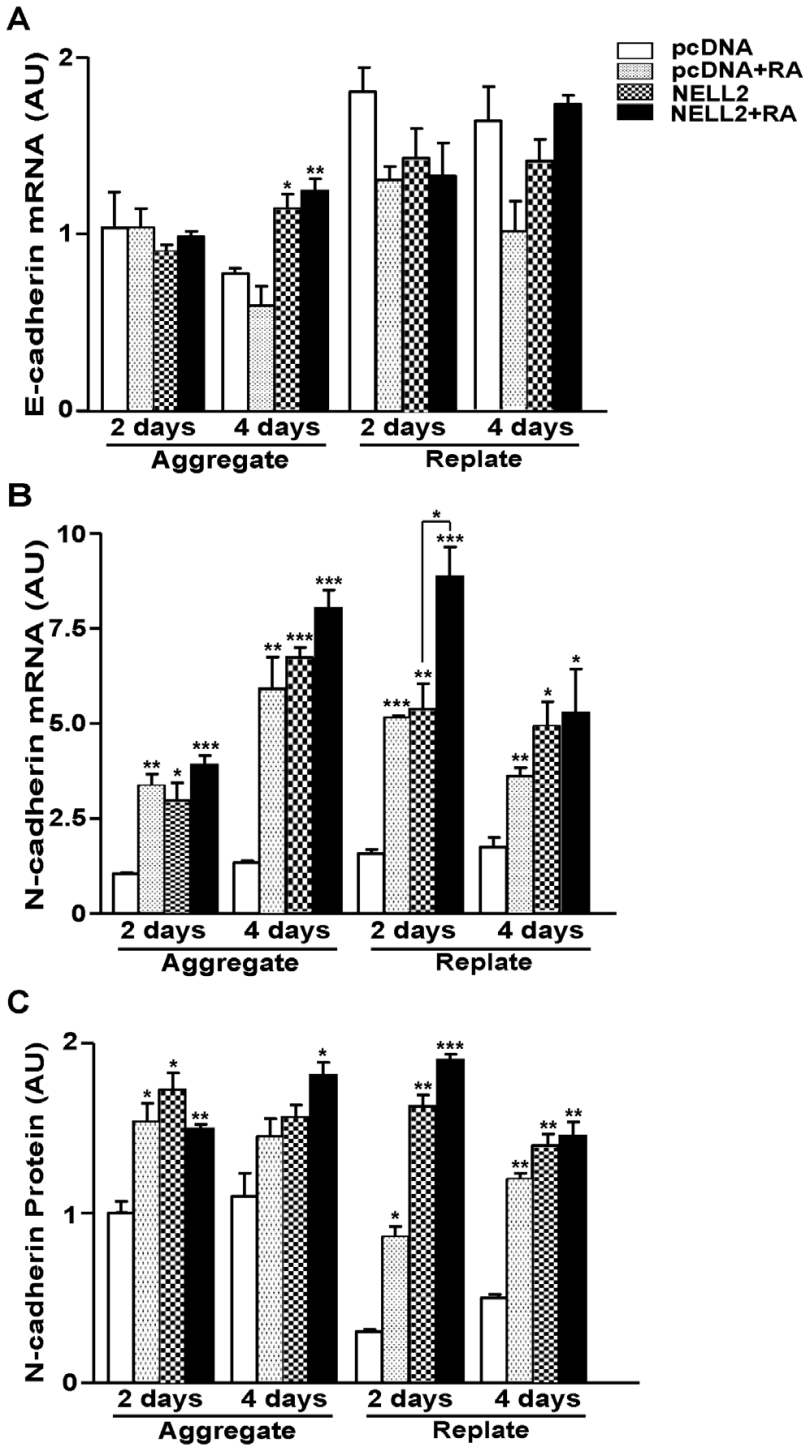
Effect of NELL2 on the N-cadherin expression in P19 cells. (A, B) Real-time PCR analysis of *E-cadherin* (A) and *N-cadherin* (B) mRNA expression in the P19 cells expressing NELL2 with or without treatment of RA, as indicated. For real-time PCR analysis, RNA samples were harvested from the cells at 2 and 4 days after aggregation and replating. (C) Data showing changes in N-cadherin protein expression calculated from Western blot analysis of samples collected at the aggregation and replating processes of neuronal differentiation of P19 cells. All experiments were repeated at least four times and data are presented as mean ± SEM. *, p<0.05; **, p<0.01; ***, p<0.001 versus control pcDNA group.

Because NELL2 induces an increase in cell aggregation, we further examined the effect of NELL2 on the expression of cadherins that are important to cell aggregation during neural induction of P19 cells [27]. Generally, overexpression of NELL2 did not induce an increase in *E-cadherin* mRNA level, except at 4 days of aggregation during the neuronal differentiation of P19 cells (Fig. 6A). RA also did not exert a significant effect on the *E-cadherin* mRNA level, as previously reported [27]. However, interestingly, NELL2 caused a significant increase in the expression of *N-cadherin* mRNA (Fig. 6B) and protein (Fig. 6C, Fig. S2) levels similar with those induced by RA [27], which suggests that NELL2 plays a role in cell aggregation during neuronal differentiation of the P19 cells via regulation of N-cadherin expression. RA treatment of cells overexpressing NELL2 further facilitated *N-cadherin* mRNA expression at 2 days after replating, suggesting that RA may affect the N-cadherin expression and cell aggregation not only through activating NELL2 expression, but also via other factors at this time point.

### *NELL2* regulates N-cadherin expression through the ERK signaling pathway

**Figure 7:**
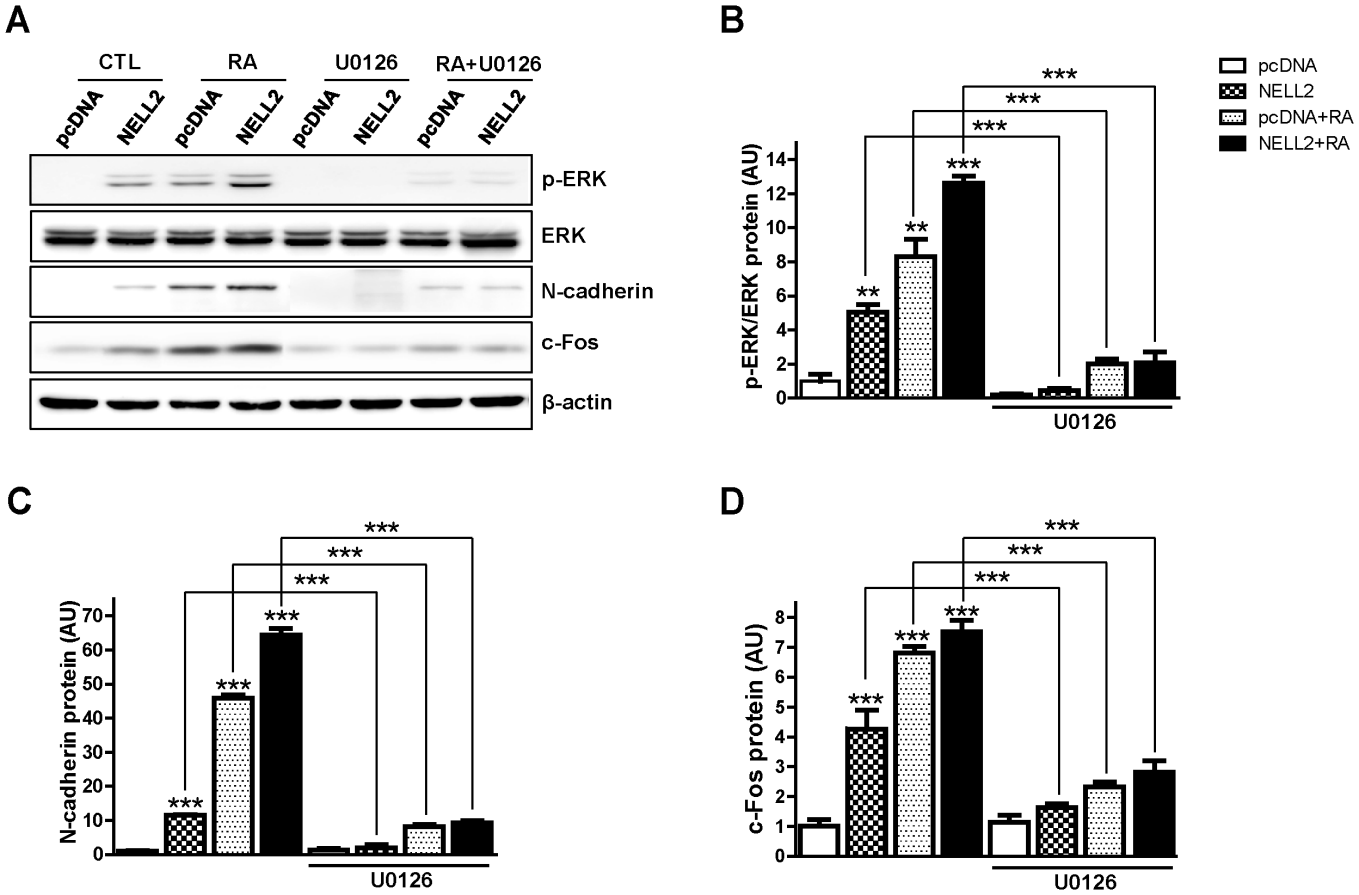
NELL2 regulates N-cadherin expression through the ERK signaling. P19 cells permanently transfected with NELL2 expression vectors were treated with 5 µM U0126 (a MEK inhibitor) in the presence or absence of 1 µM RA for 4 days. (A) Representative Western blots showing effect of U0126 on the ERK phosphorylation and N-cadherin and c-Fos expression. (B–D) Data showing the U0126-induced changes in phosphorylation of ERK (B) and expression of N-cadherin (C) and c-Fos (D), calculated from Western blot analyses. All experiments were repeated at least four times and data are presented as mean ± SEM. *, p<0.05; **, p<0.01; ***, p<0.001 versus control pcDNA group or between indicated groups.

**Figure 8:**
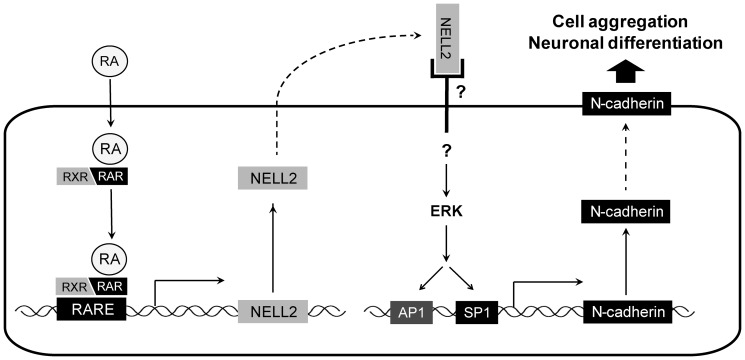
A hypothetical model for NELL2 action in the neuronal differentiation of P19 cells. RA diffuses across the plasma membrane and RA-receptor (RAR/RXR) complexes bind to the RA response elements (RAREs) of the *NELL2* promoter region. These complexes transcriptionally activate NELL2 expression, and, in turn, released NELL2 stimulates the ERK pathway via as yet unknown receptor signaling. The activated ERK increases synthesis of N-cadherin, which mediates cell-cell adhesion and neuronal differentiation.

To investigate a possible involvement of ERK signaling in the NELL2 action during the aggregation of P19 cells, the P19 cells permanently expressing NELL2 were treated with a MEK inhibitor, U0126, from day 0 to day 4 of aggregation (see Fig. 3A). The protein extracts were examined with Western blot analysis (Fig. 7A). The treatment of U0126 very effectively inhibited phosphorylation of ERK in all experimental groups (Fig. 7B). Specially, NELL2-induced ERK phosphorylation almost completely disappeared by the treatment of U0126, suggesting that NELL2 specifically induces ERK phosphorylation. Moreover, the inhibitor markedly suppressed expression of N-cadherin (Fig. 7C) as well as c-Fos (Fig. 7D) in all experimental groups. These results indicate that NELL2 stimulates the intracellular ERK signaling via yet unknown receptor and thus results in the activation of N-cadherin expression through transcriptional activator(s) of the ERK downstream such as c-Fos, an AP1 protein (see Fig. 8).

## Discussion

In this study, we found that NELL2 plays an important role in neuronal differentiation of P19 mouse embryonic carcinoma cells. Expression of the *NELL2* gene was activated by RA treatment that induces neuronal differentiation of P19 cells. RA regulation of m*NELL2* gene expression was directly mediated via binding of its receptor RAR/RXR to the binding sites on the m*NELL2* promoter region. In parallel with the neural induction after RA-induced aggregation and replating, the increase in NELL2 expression was similar to that of neuron-specific marker Tuj1. P19 cells overexpressing NELL2 revealed an increase in N-cadherin expression and increased aggregation without RA, and moreover, enhanced expression of neuronal markers after replating, suggesting an essential role of NELL2 in the aggregation and neural induction of P19 cells.

RA activates the transcription of the m*NELL2* gene through action on the two RARE sequences in the m*NELL2* promoter. Sequence analysis revealed that the m*NELL2* promoter does not contain a classical palindromic RARE. Instead, it bears two half-RAREs that show strong binding activities to RAR in EMSA and ChIP assays, suggesting that these domains physically interact with RAR. An earlier report has already shown the effectiveness of the half-RARE on the regulation of genes containing this DNA sequence [28]. Moreover, the RA action on the *NELL2* gene expression through the two half-RARE sequences was further confirmed by the disappearance of the stimulatory effect of RA on the m*NELL2* promoter activity after deleting both half-RAREs.

Aggregation in the presence of RA induces P19 cells to differentiate into the neuroectoderm lineages such as neurons and glial cells [10], [14], [15], whereas aggregation of P19 cells without the RA treatment results in the differentiation of the extraembryonic endoderm [29], [30]. However, the P19 cells without RA treatment were triggered to initiate neuronal differentiation by overexpressing some RA-induced genes such as *N-cadherin* and *Wnt-1* under aggregation inducing conditions [27], [31]. Interestingly, overexpression of NELL2, a RA-induced gene, triggered the neural differentiation of P19 cells without RA. Moreover, NELL2 increased N-cadherin expression as well as aggregation of P19 cells without RA treatment, suggesting that the effect of RA on aggregation, N-cadherin expression, and neuronal differentiation of P19 cells may be in part through NELL2 action.

One of most interesting findings of this study was induction of cell aggregation by NELL2 even without treatment with RA. Aggregation is an important initial stage in the neural induction of embryonal carcinoma cells, while the cells differentiate into neural progenitor cells [32]. RA is an inducer of this initial stage of P19 cell aggregation [33]. Our results showed that overexpression of NELL2 strongly induced the aggregation of P19 cells without RA treatment, whereas an RNA interference of the NELL2 expression completely abolished RA-induced cell aggregation, suggesting that NELL2 plays a critical role in the aggregation stage of RA-induced neuronal differentiation of P19 cells.

Both N-cadherin and neural cell adhesion molecules (NCAMs) are key factors in the control of cell aggregation (or cell-cell adhesion) and tissue morphogenesis in the nervous system [34], [35]. Cadherins have been identified as calcium-dependent homophilic cell–cell adhesion molecules [36]. The expression of N-cadherin begins with neurulation. At the midline of the embryo, the E-cadherin-positive surface ectoderm thickens to form the neural fold. The prospective neural tissue switches to express N-cadherin from E-cadherin as the lateral ridges of the neural fold fuse to form the neural tube that separates from the surface ectoderm [37]. Then the N-cadherin continuously expresses in the entire proliferative neuroepithelium during the development [38], [39].

In addition to the transcriptional regulation by RA, transcription of N-cadherin is also regulated by transcription factors, Sp1 and Ap1 [40], which are known to be activated by intracellular signaling transmitted by ERK [41], [42]. Moreover, ERK activation itself, together with N-cadherin-mediated cell aggregation, plays an important role in neuronal differentiation of human embryonic carcinoma cells, hNTera2/c.D1 (NT-2) [43]. Activation of ERK also plays an important role in RA-induced neuronal differentiation. The RA-induced neuronal differentiation of mouse embryonic stem cells (D3 ES cells) was significantly inhibited by treatment with U0126, an ERK inhibitor [44]. Moreover, RA simulated the ERK signaling pathway during neuronal differentiation of human embryonic carcinoma NT-2 cells [43]. Stimulation of ERK signaling increases *N-cadherin* gene expression via action of its downstream transcription factor(s) on the SP1 and AP1 site of the *N-cadherin* gene promoter [40]. Therefore, RA may stimulate the N-cadherin expression at least partly through the activation of ERK signaling, though the mechanism involved in RA activation of ERK signaling is not yet fully understood.

In this study, we also found that treatment with RA increased ERK phosphorylation during neuronal differentiation of P19 cells. Moreover, NELL2 alone strongly stimulated the phosphorylation of ERK in P19 cells, as it did in an earlier study using HiB5 cells, where estrogen transcriptionally activated NELL2 expression, and, in turn, the released NELL2 stimulated the ERK pathway via an as yet unknown receptor resulting in estrogen-dependent neuronal protection [13]. Similarly, in the neuronal differentiation process, RA transactivates NELL2 expression, and then NELL2 stimulates intracellular ERK signaling for the synthesis of N-cadherin, which is critical for cell-cell adhesion and differentiation of neuronal fate of P19 cells (summarized in Fig. 8).

In summary, our results indicate that, under the transcriptional control of RA, NELL2 plays an important role in the neuronal differentiation of neuroprogenitor cells by stimulating N-cadherin synthesis and, consequently, cell-cell aggregation via intracellular ERK signaling in the P19 cell system. Further study will be required to uncover the potential mechanism of NELL2 action that may generally play a critical role in the regulation of neuronal differentiation via control of N-cadherin expression and cell aggregation.

## Supporting Information

Figure S1 **Nucleotide sequence of 5′-flanking region of the m** ***NELL2*** **gene.** Nucleotides are relatively numbered by assigning the translational start site of the ATG codon at the +1 position. Bold letters represent the first and second exons, and the lowercase letters indicates the first intron. Arrows represent two transcription start sites. Underlined nucleotides are putative binding domains for transcription factors such as progesterone receptor (PR)/glucocorticoid receptor (GR) and SP1, presumed from sequence analysis (NCBI GenBank™ accession number GU290311). Boxed nucleotides indicate putative half-RA response elements (RAREs).
(TIF)

Figure S2 **Effect of NELL2 on the N-cadherin expression during the neuronal induction of P19 cells.** Western blot analysis of N-cadherin expression in the P19 cells permanently expressing NELL2 with or without treatment of RA, as indicated. Protein samples were extracted from the cells at 2 and 4 days after aggregation (A and B) and replating (C and D) and were analyzed using antibodies against N-cadherin or β-actin.
(TIF)

Table S1 **Primers used for real-time PCR analysis.**
(DOCX)
