# Supplementary figures and images for: Neural Epidermal Growth Factor-Like Like Protein 2 (NELL2) Promotes Aggregation of Embryonic Carcinoma P19 Cells by Inducing N-Cadherin Expression
Source: PLoS One. 2014 Jan 21;9(1):e85898. doi: 10.1371/journal.pone.0085898 (PMC3897553; doi:10.1371/journal.pone.0085898)

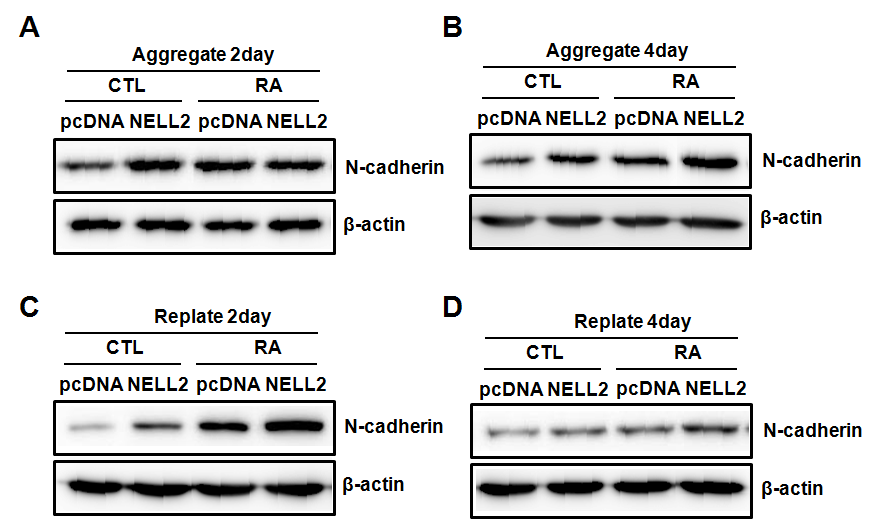

Supplement: Figure S2 — Effect of NELL2 on the N-cadherin expression during the neuronal induction of P19 cells. Western blot analysis of N-cadherin expression in the P19 cells permanently expressing NELL2 with or without treatment of RA, as indicated. Protein samples were extracted from the cells at 2 and 4 days after aggregation (A and B) and replating (C and D) and were analyzed using antibodies against N-cadherin or β-actin. (TIF) [file pone.0085898.s002.tif]
