# Supplementary material for: Neural Epidermal Growth Factor-Like Like Protein 2 (NELL2) Promotes Aggregation of Embryonic Carcinoma P19 Cells by Inducing N-Cadherin Expression
Source: PLoS One. 2014 Jan 21;9(1):e85898. doi: 10.1371/journal.pone.0085898 (PMC3897553; doi:10.1371/journal.pone.0085898)
Supplement: Table S1 — Primers used for real-time PCR analysis. (DOCX) [file pone.0085898.s003.docx]

**Table S1. Primers used for real-time PCR analysis**

| **Primer** | **Accession No.** | **Annealing**  **Temperature (^o^C)** | **Size (bp)** | **Sequences** |
| --- | --- | --- | --- | --- |
| NELL2 | NM_016743.2 | 53 | 190 | 3’-tgt aga aac gga gga gcg tg-5’  5’-ggt agc cgt ctc tgc act ca-3’ |
| E-cadherin | NM_009864.2 | 53 | 104 | 3’-cat gtt tcc cag cgt cta cc-5’  5’-tct ttg gaa att cgc cct tt-3’ |
| N-cadherin | NM_007664 | 53 | 110 | 3’-aat ggg tct gtt cca gag gg-5’  5’-ctg tac cgc agc att cca tt-3’ |
| Ngn 1 | NM_010896 | 53 | 193 | 3’-gct cac caa gat tga gac gc-5’  5-gga acc cca gga ctc agt gt-3’ |
| Nestin | NM_016701 | 53 | 178 | 3’-ggt tcc caa ggt ctc cag aa-5’  5’-aaa tgc ctg ctg gtc ctc tt-3’ |
| Tuj 1 | NM_023279 | 53 | 192 | 3’-agc aga tgt tcg atg cca ag-5’  5’-tac ctt gac gtt gtt ggg ga-3’ |
| GAPDH | NM_008084.2 | 53 | 203 | 3’-gga gcc aaa agg gtc atc at-5’  5’-gtg atg gca tgg act gtg gt-3’ |
